# Rapid Regeneration and Reuse of Silica Columns from PCR Purification and Gel Extraction Kits

**DOI:** 10.1038/s41598-018-30316-w

**Published:** 2018-08-27

**Authors:** Ying Zhou, Yang Zhang, Wei He, Juan Wang, Feixia Peng, Liyun Huang, Shasha Zhao, Wensheng Deng

**Affiliations:** College of Life Science and Health, Wuhan University of Science and Technology, Wuhan, 430065 China

## Abstract

Silica columns from PCR purification and gel extraction kits are widely used in laboratories worldwide to assist in gene cloning. However, the use of these columns can generate plastic waste that has an environmental impact due to their one-off design and massive consumption. Thus, it is important to develop a novel method that can reduce the utilization of silica columns but not affect research efficiency. In this study, various chemical and nonchemical reagents were used to eliminate residual DNA within used columns from PCR purification and gel extraction kits. We show that phosphoric acid is the most effective reagent among those tested to remove DNA contamination from used columns. Columns regenerated using 1 M phosphoric acid have a DNA purification capability that is comparable to that of fresh columns. We demonstrate that silica columns can be regenerated and reused a minimum of five times. The lab-made buffers are compatible with the regenerated columns for DNA purification, and DNA that is prepared with the regenerated columns can be used for gene cloning without affecting the gene cloning efficiency. Thus, the use of this novel method greatly reduces the production of laboratory waste and benefits numerous laboratories worldwide.

## Introduction

Molecular cloning is one of the greatest techniques developed in the 20^th^ century^1–3^ and is used in a range of research areas, including gene replication, recombinant protein preparation, the generation of transgenic organisms, gene function analyses and gene therapy^4–9^. Molecular cloning, also referred to as gene cloning, consists of a series of experiments that allow recombinant DNA molecules to be assembled and replicated in host organism. In the laboratory, gene cloning can be performed using only manual techniques or by using manual methods in combination with commercial kits^10,11^. The use of commercial kits, such as those designed for DNA extraction, PCR purification, and gel extraction, improves the quality of DNA obtained and greatly accelerates the experimental processes. However, commercial kits are made for one-off use, are expensive for most laboratories worldwide and they generate plastic waste that can have a negative implication on the environment^12,13^. Thus, the goal of this study was to develop a novel method that can reduce the utilization of commercial kits while not affecting research efficiency.

Silica resins have been used for DNA and RNA preparation for decades^14–16^, and silica columns are the key materials within commercial kits. It has been shown that silica or glass resins can be regenerated, although DNA cross-contamination was detected in early studies^17,18^. A later study demonstrated that used columns from DNA extraction kits can be repeatedly regenerated and utilized for several times without carryover contamination^19^, although this method is time-consuming. This limitation has been overcome by using a 45-minute approach developed by Tagliavia’s research group^20^. However, previous methods for column regeneration were primarily focused on plasmid or genomic DNA extraction kits^19,20^. Whether the silica columns from PCR purification kits (also called clean-up kits) and gel extraction kits can be regenerated and reused for gene cloning remains unclear. In this study, we systemically analysed the amount of residual DNA in silica columns that were cleaned using a variety of chemical and nonchemical reagents based on three different protocols. We show that phosphoric acid is the most effective reagent to eliminate residual DNA trapped in the used columns. Columns that were regenerated using 1 M phosphoric acid can purify DNA as efficiently as fresh columns and can be regenerated and reused for DNA purification a minimum of five times. The lab-made buffers are compatible with the regenerated columns and have a capability for DNA purification that is comparable to commercial buffers. We show that the quality of DNA prepared with the regenerated columns is sufficient to meet the requirements of gene cloning.

## Results

### Establishment of a regeneration method for disposable silica columns from a PCR purification kit

**Figure 1:**
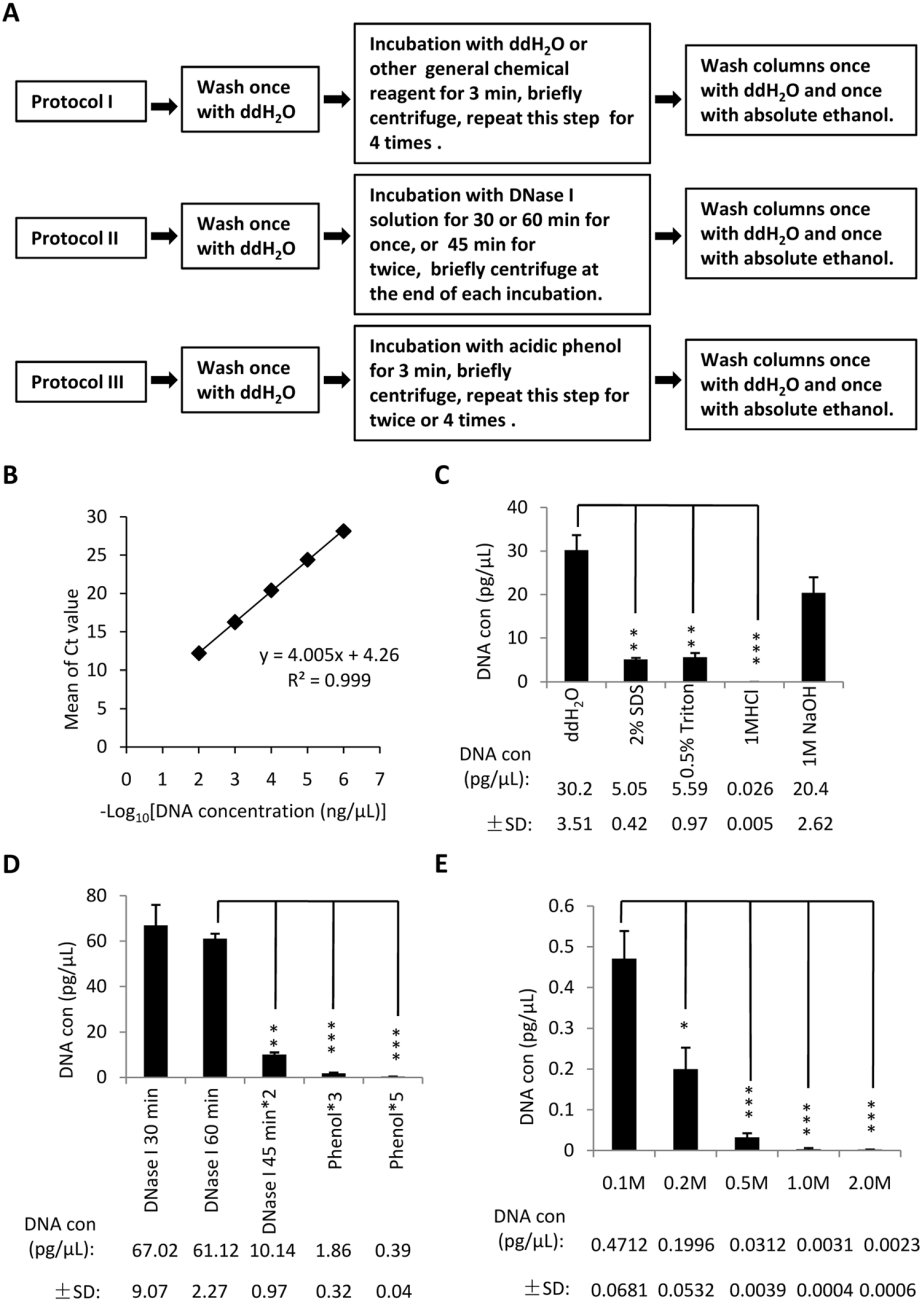
Phosphoric acid is the most effective reagent for eliminating residual DNA from used Qiagen PCR purification kit columns. (**A**) A scheme showing the workflow for different protocols used in this study. (**B**) The standard curve that was plotted with the Ct values against –Log_10_ DNA concentration (ng/μL). Ten microliters of LIF DNA template at a concentration of 100 ng/μL was consecutively diluted at 1/10 for 8 times, and the diluted samples (starting from 1 * 10^−2^ ng/μL) were used for qPCR. Ct: Threshold cycles in quantitative PCR. (**C**) qPCR analysis to assess the amount of residual DNA eluted from the columns that were cleaned with different chemical reagents. LIF-contaminated columns were cleaned with indicated reagent based on protocol I. One microlitre of eluate was used to assess the presence of residual DNA by qPCR, with each sample assayed in triplicate. The obtained Ct values were converted into DNA concentration using the standard curve in B and were subjected to statistical analysis. The value of each column within the graph is presented in the bottom of the panel to clearly show the differences between the samples. (**D**) qPCR results for the amount of residual DNA from the columns that were cleaned with DNase I or acidic phenol. Contaminated columns were cleaned based on protocol II or III. qPCR assays were performed as in C. (E) qPCR showing DNA elimination for the used columns cleaned with different concentrations of phosphoric acid. Contaminated columns were cleaned with different concentrations of phosphoric acid as detailed in protocol I. qPCR assays performed as in C. Each column in C–E represents the mean ± SD of three independent experiments. *p < 0.05; **p < 0.01 and ***p < 0.001, where *p* was obtained by a one way ANOVA.

To establish the method for regenerating used columns, fresh columns from a PCR purification kit (Qiagen) were initially used to purify a human LIF gene from PCR product. The contaminating DNA in the used columns were eliminated using general chemical reagents, including ddH_2_O, 1 M HCl, 1 M NaOH, 2% SDS and 0.5% Triton X-100 as described in protocol I (Fig. 1A). To evaluate the efficacy of the DNA elimination treatments, a standard curve was established using the mean qPCR Ct values plotted against the minus Log_10_ values of different concentrations of LIF DNA (Fig. 1B). Thirty microlitres of eluate from the regenerated columns was used to determine the amount of residual DNA. The concentration of residual DNA in the eluate was evaluated by determining the Ct values from qPCR and the standard curve. Figure 1C shows that most of the tested chemical reagents were able to effectively remove the DNA remaining in the used columns, although DNA was still detected in each sample. In particular, the approach using 7 consecutive washes with ddH_2_O was less efficient than those with 2% SDS, 0.5% Triton X-100 and 1 M HCL. The use of 1 M NaOH was slightly better than ddH_2_O, but far worse than 2% SDS, 0.5% Triton X-100 and 1 M HCL. The use of 1 M HCl was the most effective reagent for eliminating the residual DNA from the used columns among the tested reagents, as the concentration of residual DNA reduced to 0.026 pg/μL using this treatment (Fig. 1C). These data indicate that DNA within the used columns could not be completely removed using the chemical solutions in protocol I.

DNase I is often used to digest or remove contaminating DNA in biological and biochemical research^21^. However, whether DNase I could effectively remove the residual DNA from the used columns was unclear. The LIF-contaminated columns were next cleaned with a 1*DNase I solution as detailed in protocol II (Fig. 1A). The DNA remaining within the regenerated columns was eluted and detected by qPCR. Unexpectedly, the DNase I solution could not completely digest and remove the residual DNA in the used columns (Fig. 1D). The elimination of DNA from the used columns treated twice with the DNase I solution was worse than that observed using 2% SDS in protocol I (Fig. 1C,D). Although increasing the number of washes significantly reduced the amount of remaining DNA (Fig. 1D), the need to do so would also increase the cost of column regeneration. Thus, the use of DNase I is not a good option for regenerating the used columns.

Acidic phenol has been shown to effectively eliminate DNA template from a reaction mixture in a non-radioactive *in vitro* transcription assay^22^. We next performed DNA elimination assays using LIF-contaminated columns and acidic phenol as described in protocol III (Fig. 1A). The eluted DNA from the regenerated columns was analysed by qPCR. The results showed that acidic phenol significantly reduced the amount of residual DNA within the regenerated columns when compared with the DNase I-60 min assay treatment (Fig. 1D). However, the elimination of residual DNA from the used columns using acidic phenol was worse than that observed using 1 M HCl (Fig. 1C,D), suggesting that acidic phenol is not suitable for column regeneration.

The inability to completely eliminate residual DNA from the used columns prompted us to seek a novel approach. Considering that DNA molecules consist of double strands of phosphate-deoxyribose backbone and bases that contain a large number of phosphate groups, we next tested if the DNA in the used columns could be effectively removed using a phosphoric acid solution. Different concentrations of phosphoric acid were used to perform DNA elimination assays based on protocol I (Fig. 1A). Strikingly, phosphoric acid solutions showed a great capability of eliminating the contaminated DNA from the used columns (Fig. 1E). The concentration of residual DNA eluted from the used columns was significantly reduced to 0.0031 pg/μL from 0.4712 pg/μL when the concentration of phosphoric acid was increased to 1.0 M from 0.1 M. However, only a slight reduction was observed when the concentration of phosphoric acid was increased to 2.0 M from 1.0 M (Fig. 1E). The concentration of residual DNA observed using 1 M phosphoric acid was approximately 10 times lower than that observed using 1 M HCl (Fig. 1C,E) although trace amounts of DNA was still detected.

Since silica columns are typically designed to purify up to 10 μg of DNA, 50 μL of PCR product may not saturate the DNA-binding capacity of a fresh column, which could lead to underestimation of the residual DNA. Next, fresh columns were used to purify 5 or 25 μg of LIF DNA using a Qiagen PCR purification kit. The DNA-contaminated columns were regenerated with general chemical reagents, including phosphoric acid, ddH_2_O and others according to protocol I shown in Fig. 1A. The results showed that a significant difference was not observed in the concentration of residual DNA in the columns used to purify either 5 or 25 μg of DNA, although the excessive amount of DNA used for purification slightly increased the residual DNA within the restored columns (see Supplementary Fig. S1). Taken together, these data indicate that 1 M phosphoric acid is the most promising reagent for removing DNA contamination from the used columns.

### Regenerated columns possess a comparable capacity for DNA purification as fresh columns from PCR purification kits

**Figure 2:**
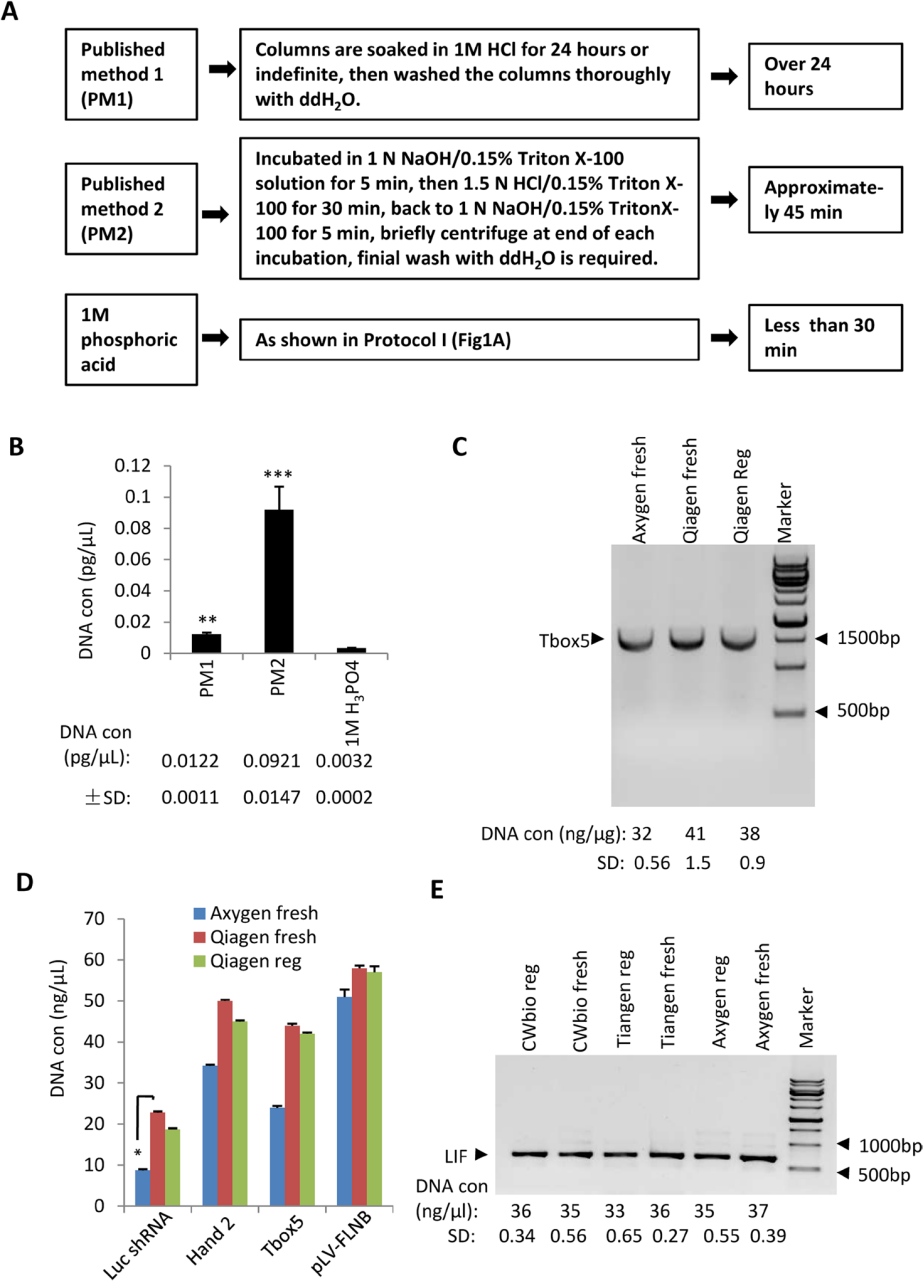
Regenerated PCR purification kit columns have a comparable capacity for DNA purification as fresh columns. (**A**) A scheme showing the workflow and duration for the published regeneration methods and for the 1 M phosphoric acid method. PM: published method. (**B**) Comparison of DNA elimination using 1 M phosphoric acid and the published methods. The used columns were cleaned with the protocol as shown in A. Thirty microlitres of eluate was obtained from the regenerated columns, 1 μL of which was used for qPCR. The data were processed as shown in Fig. 1C. (**C**) Comparison of DNA purification efficacy between the regenerated and fresh columns. A 50 μL Tbox 5 PCR reaction was purified with the regenerated or fresh columns, and the DNA concentrations were measured with a Nanophotometer (Thermo Scientific) and then subjected to a statistical analysis. The DNA quality was verified by agarose gel electrophoresis and imaged using a Bio-Rad ChemiDoc XRS + System. Data acquisition and settings are described as in the materials and methods section. The full-length image is presented in Supplementary Fig. S2. The DNA concentration for each sample is shown beneath the gel picture. (**D**) The regenerated columns showed a similar capacity for purifying different sizes of DNA as the fresh columns. DNA from a 50 μL PCR reaction was purified and quantified as in C. Luc shRNA, Luciferase shRNA cDNA, 70 bp; Hand2, 654 bp, Tbox 5, 1557 bp; pLV-FLNB, 17360 bp. (**E**) Comparison of DNA purification efficacy between the regenerated and fresh columns from different PCR purification kits. DNA from a 50 μL LIF PCR reaction was purified using regenerated or fresh columns, and the data were processed and presented as in C. Each column in B and D represents the mean ± SD of three independent experiments. *p < 0.05; **p < 0.01 and ***p < 0.001, where *p* was obtained by a one way ANOVA.

Previous studies have revealed that the used columns from DNA extraction kits for plasmid or genomic DNA preparation can be regenerated and reused for DNA extraction^19,20^. Next, LIF-contaminated columns from PCR purification kit were cleaned with two published methods (PM1^19^ and PM2^20^) or with 1 M phosphoric acid (Fig. 2A), where the residual DNA from the regenerated columns was analysed by qPCR. The results showed that both PM1and PM2 were able to effectively remove the residual DNA from the used columns, although the 0.0122 and 0.0921 pg/μL of residual DNA eluted from the PM1- and PM2-regenerated columns respectively, was significantly higher than that obtained using 1 M phosphoric acid (Fig. 2B). In addition, the time consumed using 1 M phosphoric acid was less than that for PM1 or PM2 (Fig. 2A). Thus, these data confirm that 1 M phosphoric acid is the most effective and rapid method for the regeneration of used columns.

In the laboratory, PCR products (DNA) are typically purified with PCR purification kits to assist in gene cloning. Thus, we next examined the capability of the regenerated columns to purify DNA. A 50 μL PCR reaction of Tbox 5 DNA was purified with regenerated or the fresh columns (Fig. 2C), and the resulting DNA concentrations were measured with a Nanophotometer, followed by a statistical analysis and detection by agarose gel electrophoresis. Figure 2C shows that the DNA yield obtained using the regenerated Qiagen columns was not significantly reduced compared to that obtained using fresh Qiagen columns. To verify this observation, DNA fragments of different sizes were amplified by PCR and purified as shown in Fig. 2C. The results shown in Fig. 2D confirmed that regenerated Qiagen columns showed a similar capability for DNA purification as fresh Qiagen columns. In particular, both regenerated and fresh Qiagen columns showed a better ability to purify DNA than fresh Axygen columns when a 70 bp DNA fragment (a cDNA encoding luciferase shRNA) was purified (Fig. 2D). In addition, similar assays were also performed using regenerated columns from PCR purification kits made by different companies, including Axygen, Tiangen and CWBiotech. The results were consistent with those observed using the regenerated Qiagen columns (Fig. 2E). Taken together, the results show that regenerated columns from PCR purification kits can be used for DNA purification and do not affect the efficiency of DNA purification.

### Regeneration of used columns from gel extraction kits and determination of DNA purification efficacy

**Figure 3:**
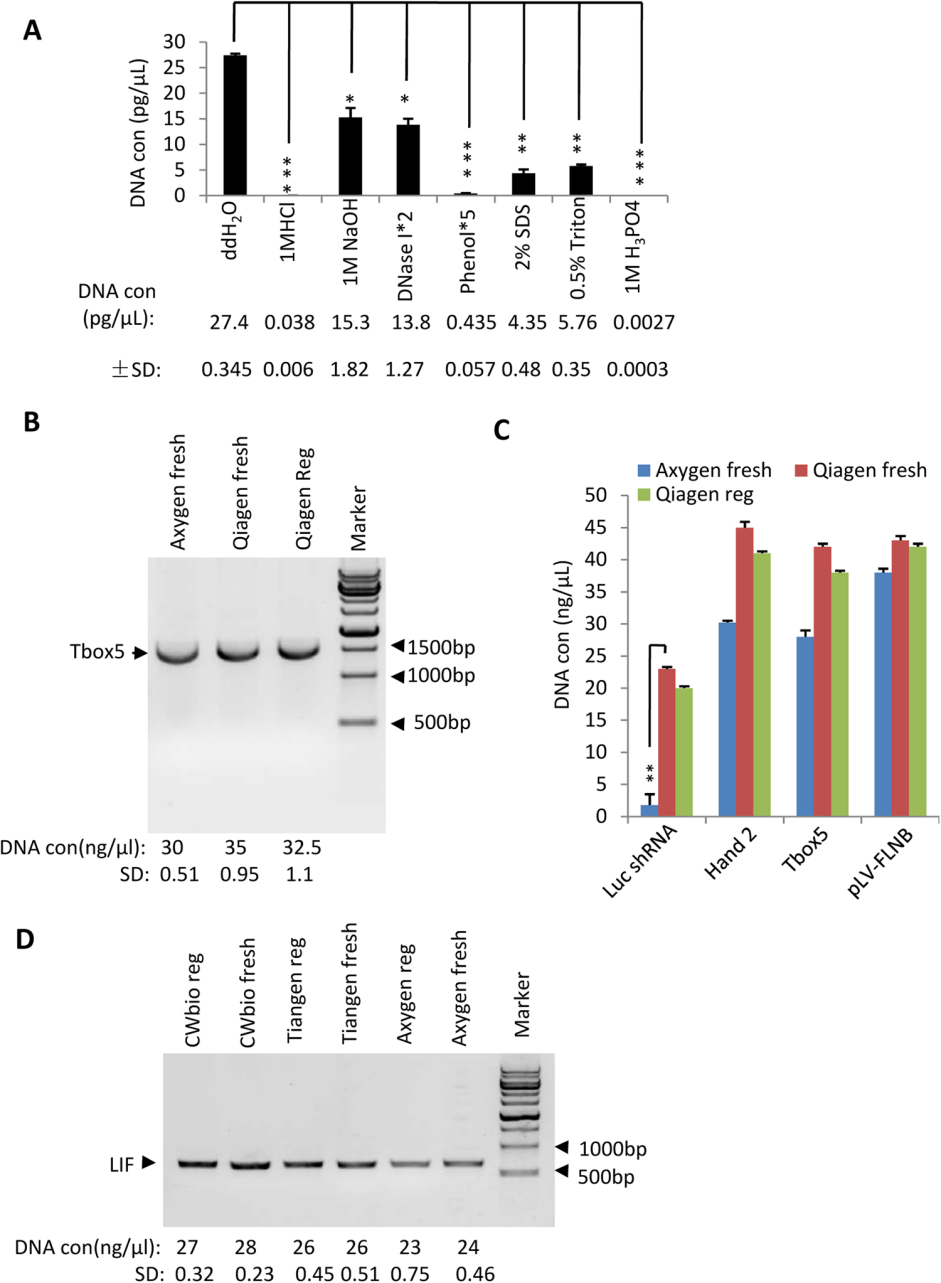
Analysis of DNA extraction efficacy using regenerated columns from gel extraction kits. (**A**) qPCR analysis for the residual DNA from Qiagen columns regenerated using different reagents. LIF-contaminated columns were cleaned with the indicated reagents based on the protocols in Fig. 1A. Thirty microlitres of eluate was obtained from the regenerated columns, 1 μL of which was used for qPCR. The data were processed and presented as in Fig. 1C. (**B**) Comparison of the DNA extraction efficacy between the regenerated and fresh columns from gel extraction kits. A 50 μL PCR reaction was used for agarose gel electrophoresis, and DNA extraction was performed using the regenerated or fresh columns according to the gel extraction kit manual. The extracted DNA was quantified with a Nanophotometer and verified by agarose gel electrophoresis as shown in Fig. 2C. The full-length image is presented in Supplementary Fig. S3. (**C**) The regenerated columns showed a comparable ability to purify different sizes of DNA as the fresh columns. A 50 μL PCR reaction was used for agarose gel electrophoresis, which was purified and measured as in (**B**). (**D**) Comparison of DNA purification efficacy between the regenerated and fresh columns from different commercial kits. A 50 μL LIF PCR reaction was used for agarose gel electrophoresis, and the DNA was extracted from the gel with regenerated or fresh columns from different gel extraction kits. The data were processed and presented as in B. Each column in A and C represents the mean ± SD of three independent experiments. *p < 0.05; **p < 0.01 and ***p < 0.001, where *p* was obtained by a one way ANOVA.

So far, we have showed that the regenerated columns from PCR purification kits have a comparable capability for DNA purification as fresh columns. We next asked whether this result could be extended to gel extraction kits. To address this question, DNA-contaminated columns from a Qiagen gel extraction kit were cleaned using the protocols described in Fig. 1A. The efficacy of DNA decontamination using different reagents was analysed as shown in Fig. 1C–E. As expected, the concentrations of residual DNA from the regenerated columns were similar to those observed in Fig. 1C–E (Fig. 3A), where 1 M phosphoric acid was still the best reagent for removing residual DNA from the used columns among the tested reagents (Fig. 3A). We next assessed the effectiveness of the columns regenerated by 1 M phosphoric acid in extracting DNA from agarose gel. DNA gel extraction was performed according to the Qiagen or Axygen manufacturer’s handbooks. Figure 3B illustrates that the regenerated Qiagen columns showed a similar DNA extraction capability as the fresh Qiagen columns. To confirm this observation, DNA fragments of different sizes were purified with the columns as shown in Fig. 3B. Both fresh and regenerated Qiagen columns displayed a similar capacity for DNA extraction (Fig. 3C) and were more effective than fresh Axygen columns when a 70-bp DNA fragment was purified (Fig. 3C). The results obtained using kits from different companies were consistent with those observed using regenerated Qiagen columns (Fig. 3D). Taken together, these data indicate that regenerated columns from gel extraction kits can be used for DNA gel extraction and do not significantly affect DNA yield.

### Disposable columns from PCR purification and gel extraction kits can be regenerated and utilized a minimum of five times

**Figure 4:**
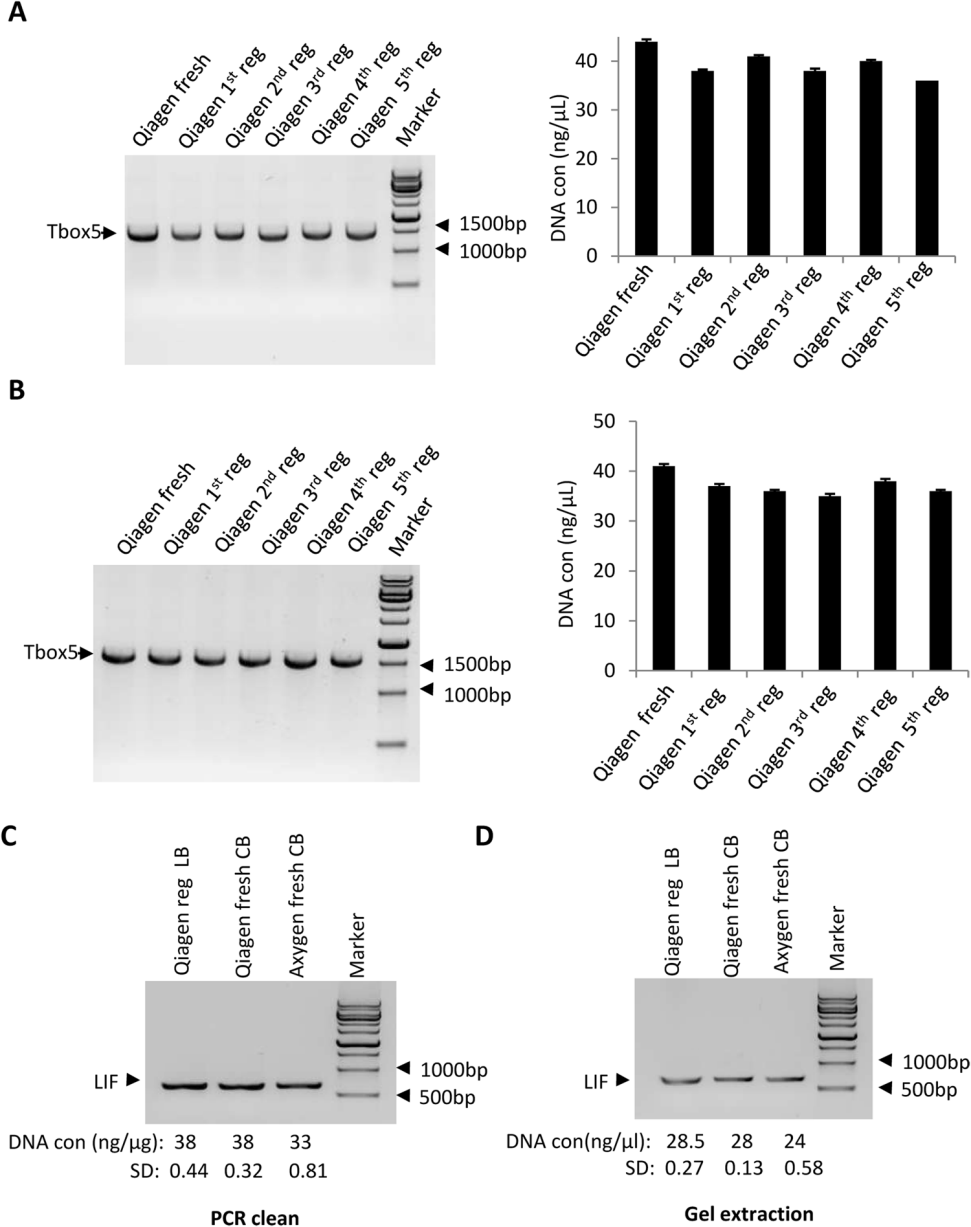
Disposable columns from Qiagen PCR purification and gel extraction kits can be repeatedly regenerated and reused a minimum of five times. (**A**) DNA purification efficacy for the repeatedly regenerated PCR purification kit columns. DNA-contaminated columns were cleaned with 1 M phosphoric acid and used for PCR product (TBox 5) purification; the same columns were regenerated and reused four additional times. The concentration of obtained DNA for each cycle was measured with a Nanophotometer and the data are presented as gel pictures (left) and in a graph (right). (**B**) The analysis of DNA extraction efficacy for the repeatedly regenerated gel extraction kit columns. DNA-contaminated columns were regenerated as in A, where a 50 μL PCR reaction was used for agarose gel electrophoresis. DNA extraction from agarose gel was performed according to the gel extraction kit manual. The data were processed as in A. The full length images for A (left) and B (left) are presented in Supplementary Fig. S4A,B. (**C**) Comparison of DNA purification efficacy between lab-made and commercial buffers. The lab-made buffers (LB) were used for DNA purification for regenerated Qiagen columns, whereas the commercial buffers (CB) were used for DNA purification for the fresh Qiagen or Axygen kit columns. The assays were performed as shown in Fig. 2C. (**D**) Comparison of DNA extraction efficacy between lab-made and commercial buffers. The lab-made buffers (LB) were used for DNA extraction from agarose gel using regenerated Qiagen columns, whereas the commercial buffers (CB) were used for DNA extraction from agarose gel using fresh Qiagen or Axygen kit columns. The assays were performed as shown in Fig. 3B. The protocol for DNA purification with lab-made buffers is described in the materials and methods section. Each column in A (right) and B (right) represents the mean ± SD of three independent experiments.

To understand whether the disposable columns from PCR purification or gel extraction kits can be repeatedly regenerated and used for DNA purification, DNA-contaminated columns from Qiagen PCR purification kits were cleaned with 1 M phosphoric acid as described in protocol I (Fig. 1A). The regenerated columns were then used to purify Tbox 5 DNA. After elution, the used columns were repeatedly regenerated and used for Tbox 5 DNA purification 4 additional times, with the concentration of DNA after each round quantified and assessed by gel electrophoresis. Figure 4A shows that the columns regenerated for the fifth time displayed a comparable DNA yield as the fresh columns, indicating that the disposable PCR purification kit columns can be recycled a minimum of 5 times. Using the same strategy shown in Fig. 4A, DNA-contaminated columns from a Qiagen gel extraction kit were repeatedly regenerated a total of 5 times and were used for DNA gel extraction between each round. Consistent with the result obtained using PCR purification kit regenerated columns, after five regenerations the columns had a similar ability to extract DNA as fresh columns (Fig. 4B).

Our data demonstrated that silica columns can be repeatedly regenerated and reused for PCR product purification or DNA gel extraction. However, the volumes of the buffers included in PCR purification or gel extraction kits are fixed, hindering the utilization of regenerated columns. Since chaotropic agents have been used for DNA or RNA extraction for decades^14,23^, we next tested whether lab-made buffers could be used for PCR product purification and DNA gel extraction. Figure 4C,D show that the DNA yields obtained using lab-made buffers and regenerated Qiagen columns was similar to those obtained using fresh Qiagen and Axygen columns. Thus, the lab-made buffers are compatible with the regenerated columns when used for PCR product purification or DNA gel extraction.

### The quality of DNA prepared with regenerated columns meets the requirements of gene cloning

**Figure 5:**
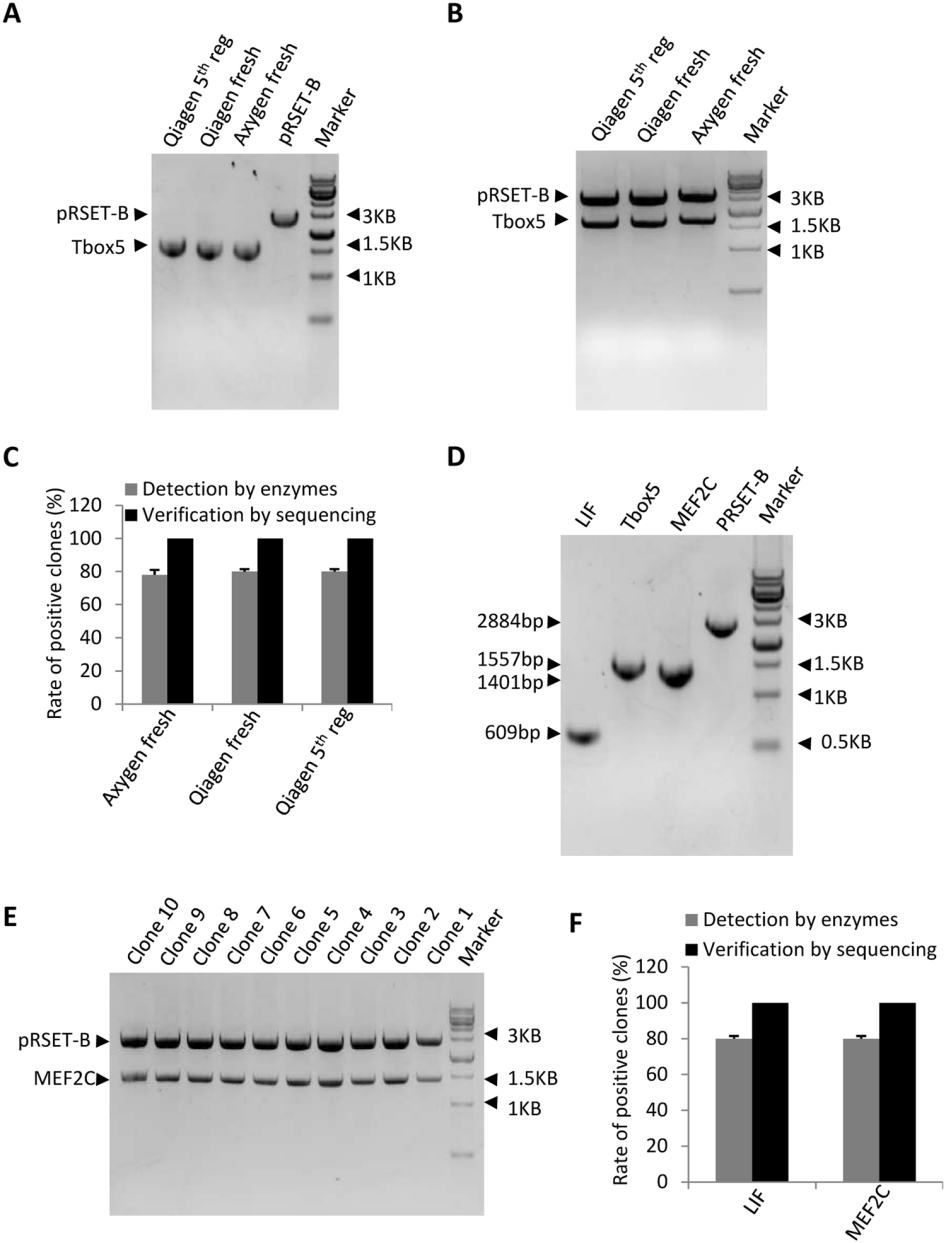
DNA prepared with the regenerated columns does not affect cloning efficiency. (**A**) Agarose gel electrophoresis image for the DNA samples purified with the regenerated and fresh columns. One microgram of DNA was digested with *Bam*HI and *Hind*III, followed by agarose gel electrophoresis and DNA extraction. (**B**) Electrophoresis image for the positive clones digested with *Bam*HI and *Hind*III. The DNA purified with the fresh or regenerated columns was used for gene cloning. Fifty colonies were picked from LB plates for plasmid preparation. Positive clones were detected by digestion with *Bam*HI and *Hind*III, and one positive clone for each sample was loaded into an agarose gel for electrophoresis. (**C**) The preparation of DNA using regenerated columns does not affect cloning efficiency. The number of positive clones obtained in B was used for statistical analysis; the positive clones were verified by DNA sequencing. (**D**) Electrophoresis image for the DNA samples purified with the regenerated columns. Tbox 5 DNA digested with *Bam*HI and *Hind*III was purified from agarose gels with the regenerated columns. The Tbox 5-contaminated columns were further regenerated with 1 M phosphoric acid and used to purify the MEF2C gene, which was also digested with *Bam*HI and *Hind*III. The obtained DNA was detected by agarose gel electrophoresis. (**E**) Electrophoresis image for MEF2C-positive clones digested with *Bam*HI and *Hind*III. Positive clones were screened as in B, and 10 positive clones digested with restriction enzymes were loaded into an agarose gel for electrophoresis. (**F**) The small amount of contaminating DNA has no chance of be cloned into the plasmid. The number of positive clones was analysed as in C. The full-length images for A, B, D and E are presented in Supplementary Fig. S5A, B, D and E. Each column in C and F within the graph represents the mean ± SD of three independent experiments.

Since the regenerated columns still carry trace amounts of DNA from the previous experiment, we next asked if the trace DNA interfered with the efficiency of gene cloning. To this end, enzyme-digested Tbox 5 DNA fragments and plasmid pRSET-B were purified with either fresh or regenerated columns from gel extraction kits (Fig. 5A). The purified Tbox 5 DNA was then ligated with the plasmid pRSET-B. Positive clones were screened by enzyme digestion and verified by DNA sequencing. As expected, the Tbox 5 DNA purified with columns that were regenerated five times was successfully cloned into the plasmid pRSET-B (Fig. 5B). The number of positive clones obtained using regenerated columns was similar to that obtained using fresh columns (Axygen or Qiagen). DNA sequencing confirmed the sequences of all positive clones as correct (Fig. 5C), indicating that the DNA prepared using regenerated columns can be used for gene cloning.

Because the regenerated columns used in the above assay were derived from the LIF*-*contaminated columns, where the LIF DNA had not been digested with restriction enzymes and the size of LIF DNA fragment was distinct from that of Tbox 5, the contaminating LIF DNA could not be cloned into pRSET-B. Thus, we next tested if residual DNA could be accidentally cloned into the plasmid pRSET-B when the size of the contaminating DNA is similar to that of target DNA and they were both digested with the same restriction enzymes. To do so, pRSET-B and the LIF and MEF2C genes were digested with *Bam*HI and *Hind*III. MEF2C has a similar size as Tbox 5 and was purified with regenerated columns carrying trace amounts of Tbox 5 DNA, which had also been digested with *Bam*HI and *Hind*III. In this assay, LIF was used as positive control and was purified using fresh Qiagen columns along with pRSET-B(Fig. 5D). DNA cloning for LIF and MEF2C was performed as shown in Fig. 5B (Fig. 5E) and analysed as shown in Fig. 5C. The data demonstrated that the number of positive clones for MEF2C was similar to that for LIF, and the sequences of all positive clones were verified as correct by DNA sequencing (Fig. 5F). Taken together, these data further confirm that the contaminating DNA from the regenerated columns has little chance to be cloned into the plasmid, suggesting that the quality of DNA prepared with the regenerated columns can meet the requirements of gene cloning.

## Discussion

Previous studies have shown that disposable columns from DNA extraction kits for plasmid preparation do not carry over contaminated DNA from previous experiments^19,20^. However, in this study, residual DNA was able to be detected by qPCR when PCR purification kit columns were regenerated by PM1 and PM2 (Fig. 2B). The discrepancy between the observations of the present and previous studies is likely attributable to the difference in the respective qPCR and autoradiography techniques used^19,20^. Apparently, qPCR is more sensitive than autoradiography for detecting residual DNA. Since the key material of columns consists of silica membranes, it is possible that the used columns from plasmid preparation kits may also be cleaned with 1 M phosphoric acid and utilized for PCR product purification or DNA gel extraction. The regenerated columns may not suitable for plasmid preparation when subsequent transfection assays are required because the results from the assays would be incorrect if trace amounts of contaminated DNA were carried over.

Although phosphoric acid has an excellent capability of eliminating DNA from the used columns, 0.0031 pg/μL of residual DNA was still detected in the eluate of the columns regenerated with 1 M phosphoric acid (Fig. 1E). It is well known that at least dozens of nanograms per microlitre of DNA are needed for ligation reactions during gene cloning^24^, which is approximately ten million times higher than the concentration of residual DNA detected from the regenerated columns. The contaminated DNA actually acts as an extremely low background during gene cloning (the signal-to-noise ratio is approximately 1 * 10^7^). Thus, there is almost no chance for the contaminated DNA to be ligated with the plasmid of interest. Indeed, in this study, contaminating Tbox 5 DNA was not detected in any positive clones when MEF2C, a similar-sized gene as Tbox 5, was digested with the same restriction enzymes and cloned into the same vector (Fig. 5F). These data suggest that trace amounts of contaminating DNA does not affect the efficiency of gene cloning, even if two similar-sized DNA fragments cannot be separated by electrophoresis, and the contaminating DNA is carried over during DNA gel extraction.

We show that phosphoric acid is the most suitable reagent for eliminating contaminating DNA among all of the tested reagents, since it could compete with DNA to bind to the silica membrane. Since the used columns can be repeatedly regenerated and reused for DNA purification, and lab-made buffers can be used for DNA purification, the use of regenerated columns can reduce the production of lab waste and help save funds for laboratories worldwide by reducing frequency at which kits need to be purchased, particularly in laboratories that routinely perform cloning. In addition, phosphoric acid is a cheap chemical reagent, and the used columns can be regenerated within 30 min. Thus, the novel regeneration method developed in this study is rapid, low-cost and can be easily applied to a range of research fields, including biology, medicine, agriculture and industry.

## Materials and Methods

### Plasmids and reagents

Human LIF, mouse Hand2, T-box 5 and MEF2C genes were cloned into the plasmid pRSET-B. A luciferase shRNA cDNA fragment was cloned into the plasmid pLV-U6-EGFP. Restriction enzymes, T4 DNA ligase and qPCR kits were purchased from Thermo Scientific Co. PCR purification and gel extraction kits were obtained from Qiagen, Axygen (Corning), Tiangen and CWBiotech, and DH5α competent cells were purchased from Bio Swamp Co. Chemicals were purchased from the Chinese Chemical Reagent Co.

### PCR and qPCR

PCR was performed using 0.5 ng of plasmid containing LIF or another genes as DNA templates in a 50 μL PCR reaction mixture containing 25 μL of Takara PCR Master Mix (2×) and 1 μL of 25 μM forward and reverse primers. PCR was performed for 35 cycles in thermal cycler (GeneAtlas) using cycling conditions that were modified based on the gene being amplified. PCR products were purified using regenerated or fresh columns and buffers from commercial clean-up kits and then were quantified with a NanoDrop 2000 spectrophotometer (Thermo Fisher). Quantitative PCR was performed using SYBR Green (Roche) and a real time detection system (Bio-Rad). qPCR data were analysed with Bio-Rad CFX Manager 3.1.

### Residual DNA elimination and column regeneration

Regenerated columns were obtained by eliminating residual DNA from the used columns. In this study, the used columns were obtained from a Qiagen PCR purification and gel extraction kit or from other commercial kits that had been used once to purify LIF or other DNA. The elimination of residual DNA from the used columns was performed using three major protocols that were specifically designed for this study. These protocols are detailed as follows:

Protocol I involves DNA elimination using generally available chemicals, including ddH_2_O, 1 M HCl, 1 M NaOH, 2% SDS, 0.5% Triton X-100 and different concentrations of a phosphoric acid solution. These chemical reagents were individually used to clean DNA-contaminated columns via the following steps: 1) 600 μL ddH_2_O was added into the used silica columns from either PCR purification or gel extraction kits, followed by centrifugation at 12,000 rpm for 30 sec. Subsequently, the waste liquid within the collection tube was discarded. 2) Six hundred microlitres of ddH_2_O or one of other chemical solutions was then added into individual columns, followed by an incubation at room temperature for 3 min. The columns were then subjected to centrifugation at 12,000 rpm for 30 sec, after which the flow-through in the collection tubes was discarded. 3) The columns were repeatedly incubated and centrifuged for 4 times as described in step 2. 4) The columns were rinsed with ddH_2_O as described in step 1. 5) The columns were washed once with absolute ethanol, completing the regeneration of the used columns (Fig. 1A).

Protocol II involves DNA elimination using DNase I. Briefly, after the used columns from commercial kits were washed with ddH_2_O, 600 μL of DNase I solution (Thermo Scientific) was added into individual columns, followed by one or two incubations at 37 °C for varied durations. After each incubation, a brief centrifugation was carried out and the flow-through was discarded. Residual DNA was eliminated by washing the columns once with ddH_2_O and once with absolute ethanol as described in protocol I (Fig. 1A).

Protocol III involves DNA elimination using acidic phenol. Acidic phenol was prepared as described previously^22^. This protocol is similar to Protocol I, but the used columns here were washed 3 or 5 times using acidic phenol (Fig. 1A).

To evaluate the elimination of residual DNA using various solutions, 30 μL of TE was added to the centre of each regenerated column, and the DNA remaining within the column was eluted by centrifugation and collected in a 1.5 mL microcentrifuge tube. One microlitre of eluted solution was used to perform qPCR, and the concentration of residual DNA was analysed based on the resulting Ct value and standard curve. The concentration of DNA was used to evaluate the elimination of residual DNA.

### DNA purification with lab-made buffers

Five volumes of DNA binding buffer (3 M NaCl, 30% ethanol) was added to one volume of PCR reaction product. The sample was mixed thoroughly by vortexing and transferred into a regenerated Qiagen column with a collection tube. The column was centrifuged at 12,000 rpm for 30 sec, after which the flow-through was discarded. Next, 600 μL of washing buffer (80% ethanol, 10 mM Tris-HCl (pH 8.0), 100 mM NaCl and 1 mM EDTA) was added to the column, which was then centrifuged at 12,000 rpm for 30 sec and the flow-through was removed. The empty column was centrifuged once more for 1 min, after which 30 μL of ddH_2_O was added to the centre of the column and was eluted into a 1.5 mL sterile microtube by centrifuging at 12,000 rpm for 1 min. The DNA concentration of the eluate was determined using a Nanophotometer (Thermo Scientific). For DNA extraction from agarose gel, after electrophoresis, the band containing the target DNA was cut out from the gel and weighed in a 1.5 mL microtube. Three volumes of gel dissolving buffer (6 M guanidine hydrochloride, 25 mM sodium citrate, 100 mM N-lauroylsarcosine, 1 mM 2-mercaptoethanol, pH 6.5) was added to one volume of gel (100 mg gel≈100 μL). The microtube containing the gel and buffer was incubated in a 75 °C heat block until the agarose gel was completely dissolved. Next, one volume of isopropanol (approximately equivalent to gel weight) was added to the sample. After vortexing, the sample was transferred to a Qiagen regenerated column and centrifuged at 12,000 rpm for 1 min, followed by wash, elution and quantification steps as performed for the PCR product purification.

### Gene cloning and sequencing

The LIF, Tbox 5 and MEF2C genes were amplified with a PCR kit (Takara Co.) from their respective plasmids. The resulting PCR products were purified with regenerated or fresh columns and using Qiagen or Axygen PCR purification kit buffers. The purified PCR products and the plasmid pRSET-B were then digested with the restriction enzymes *Bam*HI and *Hind*III. The DNA digestion mixture was subjected to agarose gel electrophoresis for 20 minutes, after which the band containing the DNA fragments was cut out, weighed and dissolved in gel extraction buffers. Next, the DNA was purified using regenerated or fresh columns and the buffers from commercial gel extraction kits. The recovered DNA was used for ligation, followed by transformation, which were performed according to standard procedures^10^. Positive clones were detected by digestion with *Bam*HI and *Hind*III, and the sequences were verified by DNA sequencing, which was performed by Tsingke Co. (Wuhan, China).

### Data acquisition and settings

Agarose gel electrophoresis was performed at 90 V for 20 min, after which the gel was imaged using a Bio-Rad ChemiDoc XRS + System with following a nucleic acid ethidium bromide staining protocol. The exposure time was set for 0.5 sec; the image colour was set grey and inverted presentation.

## Electronic supplementary material

Supplementary Information

## References

[1] Jackson D, Symons R, Berg P (1972). Biochemical method for inserting new genetic information into DNA of Simian Virus 40: Circular SV40 DNA molecules containing lambda phage genes and the galactose operon of Escherichia coli. Proc. Natl. Acad. Sci. USA.

[2] Lobban P, Kaiser A (1973). Enzymatic end-to end joining of DNA molecules. J. Mol. Biol..

[3] Cohen S, Chang A, Boyer H, Helling R (1973). Construction of biologically functional bacterial plasmids *in vitro*. Proc. Natl. Acad. Sci. USA.

[4] Nathans D, Smith HO (1975). Restriction endonucleases in the analysis and restructuring of DNA molecules. Ann. Rev. Biochem.

[5] Johnson IS (1983). Human insulin from recombinant DNA technology. Science.

[6] Lahlou H, Muller WJ (2011). β1-integrinssignalling and mammary tumor progression in transgenic mouse models: implications for human breast cancer. Breast Cancer Res..

[7] Horii T (2017). Efficient generation of conditional knockout mice via sequential introduction of lox sites. Sci. Rep..

[8] Herrera-Carrillo E, Berkhout B (2015). The impact of HIV-1 genetic diversity on the efficacy of a combinatorial RNAi-based gene therapy. Gene Ther..

[9] Min HJ, Jung YJ, Kang BG, Kim WT (2016). CaPUB1, a Hot Pepper U-box E3 Ubiquitin ligase, confers enhanced cold stress tolerance and decreased drought stress tolerance in transgenic rice (Oryza sativa L.). Mol. Cells..

[10] Russell, D. W. & Sambrook J. Molecular cloning: a laboratory manual. Cold Spring Harbor N.Y. *cold spring Harbor Laboratory* (2001)

[11] Trigoso YD, Evans RC, Karsten WE, Chooback L (2016). Cloning, Expression, and Purification of Histidine-Tagged Escherichia coli Dihydrodipicolinate Reductase. PLoS One..

[12] North EJ, Halden RU (2013). Plastics and Environmental Health: The Road Ahead. Rev. Environ. Health..

[13] Barnesl DKA, Galgani F, Thompson RC, Barlaz M (2009). Accumulation and fragmentation of plastic debris in global environments. Phil. Trans. R. Soc. B..

[14] Matitashvili E, Zavizion B (1977). One tube extraction of DNA or RNA from agarose gel. Anal. Biochem..

[15] Tian H, Hühmer AF, Landers JP (2000). Evaluation of silicaresins for direct and efficient extraction of DNA from complex biological matrices in a miniaturized format. Anal. Biochem..

[16] Dederich DA (2002). Glass bead purification of plasmid template DNA for high throughput sequencing of mammalian genomes. Nucleic Acids Res..

[17] Fogel BL, McNally MT (2000). Trace contamination following reuse of anion-exchange DNA purification resins. BioTechniques..

[18] Kim AI, Hebert SP, Denny CT (2000). Cross-contamination limits the use of recycled anion exchange resins for preparing plasmid DNA. BioTechniques..

[19] Siddappa NB, Avinash A, Venkatramanan M, Ranga U (2007). Regeneration of commercial nucleic acid extraction columns without the risk of carryover contamination. BioTechniques..

[20] Tagliavia M, Nicosia A, Gianguzza F (2009). Complete decontamination and regeneration of DNA purification silica columns. Anal. Biochem..

[21] Jahn CE, Charkowski AO, Willis DK (2008). Evaluation of isolation methods and RNA integrity for bacterial RNA quantitation. J. Microbiol. Methods..

[22] Wang J, Zhao S, Zhou Y, Wei Y, Deng W (2015). Establishment and validation of a non-Radioactive method for in vitrotranscription assay using primer extension and quantitative real time PCR. PLoS One..

[23] Lakshmi R, Baskar V, Ranga U (1999). Extraction of superior-quality plasmid DNA by a combination of modified alkaline lysis and silica matrix. Anal. Biochem..

[24] Ausubel, F. M. *et al*. Short protocols in molecular biology (the fifth edition), 3–32 (John Wiley & Sons Inc, 2002)

