# Supplementary material for: Rapid Regeneration and Reuse of Silica Columns from PCR Purification and Gel Extraction Kits
Source: Sci Rep. 2018 Aug 27;8:12870. doi: 10.1038/s41598-018-30316-w (PMC6110862; doi:10.1038/s41598-018-30316-w)
Supplement: Supplementary file 1 — Supplementary Information [file 41598_2018_30316_MOESM1_ESM.pdf]

# Rapid Regeneration and Reuse of Silica Columns from PCR Purification and Gel Extraction Kits

Ying Zhou#, Yang Zhang#, Wei He#, Juan Wang, Feixia Peng, Liyun Huang, Shasha Zhao*, Wensheng Deng*

College of Life Science and Health, Wuhan University of Science and Technology, Wuhan, 430065, China

# : These authors contributed equally to this work

*: Address correspondence to: Wensheng Deng, dengwensheng@wust.edu.cn and Shasha Zhao, zhaoshasha@wust.edu.cn .

# Supplementary information

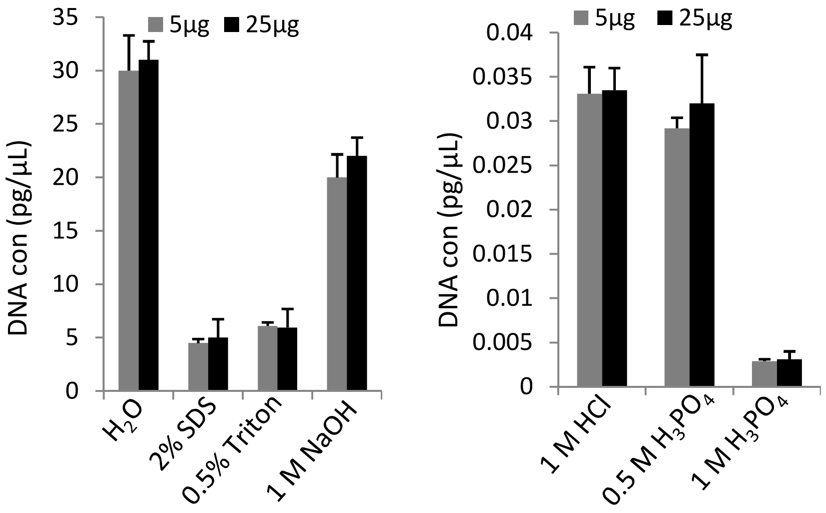


**Fig S1. Comparison of the amount of residual DNA between the regenerated columns that were used to purify 5 or 25 µg DNA before regenerating columns .** Fresh columns were used to purify 5 or 25 µg of cleaned *LIF* DNA with Qiagen purification kit, the DNA-contaminated columns were respectively cleaned with general chemical reagents (left panel) , HCl (right panel) and $H_3PO_4$ (right panel) according to the Protocol I in Fig 1A. Thirty microlitres of TE was added into the regenerated column and eluted by centrifugation. One microlitre of eluate was used for qPCR and each sample was assayed in triplicates, the resulting Ct values were used to determine DNA concentration using the standard curve in Fig 1B and subjected to statistical analysis.

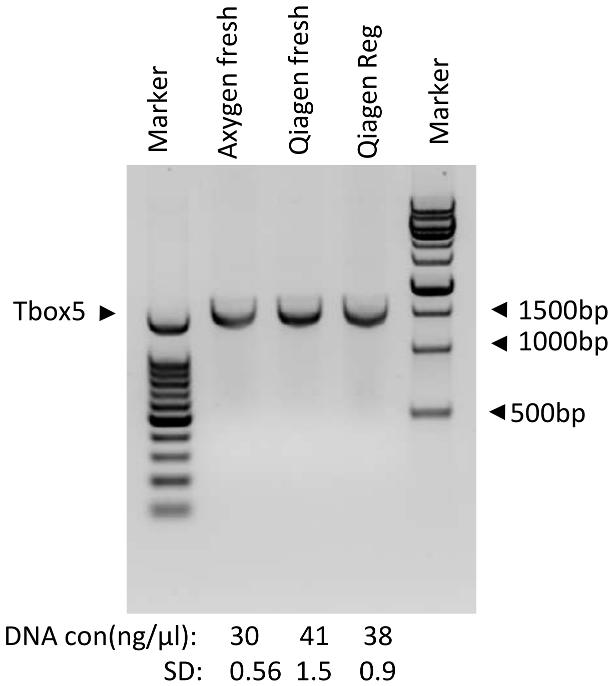


**Fig. S2. Comparison of DNA purification efficacy between the regenerated and fresh columns**. The DNA from a 50 µL PCR product was purified with the regenerated or fresh columns; DNA concentration for individual sample was measured with Nanophotometer, subjected to statistical analysis. DNA quality was verified by agarose gel electrophoresis and then imaged under the Bio-Rad ChemiDoc XRS+ System, data acquisition and setting are described as in Material and Methods. The DNA concentration for each sample is shown beneath the gel.

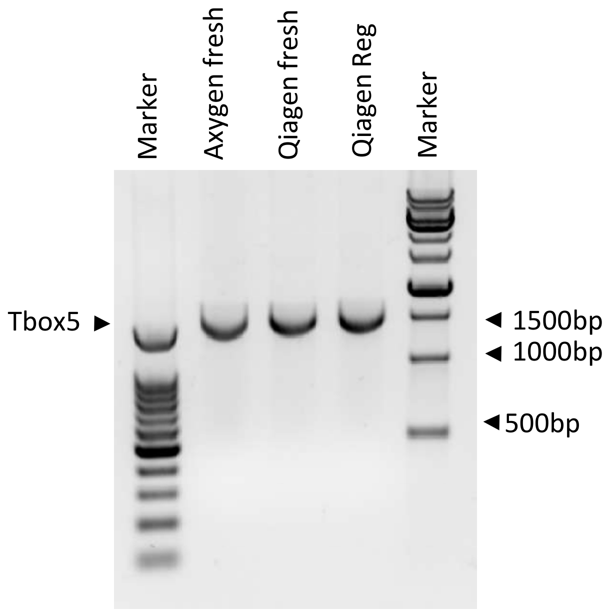


DNA con(ng/µl): 30 35 32.5
SD: 0.51 0.95 1.1

**Fig. S3. Comparison of DNA extraction effect between the regenerated columns and the fresh columns from gel extraction kits**. Fifty microlitres of PCR product was loaded in an agarose gel for electrophoresis, DNA was extracted using the regenerated or fresh columns according to the manual of gel extraction kit, quantified with Nanophotometer and verified by agarose gel electrophoresis as shown in Fig S2.

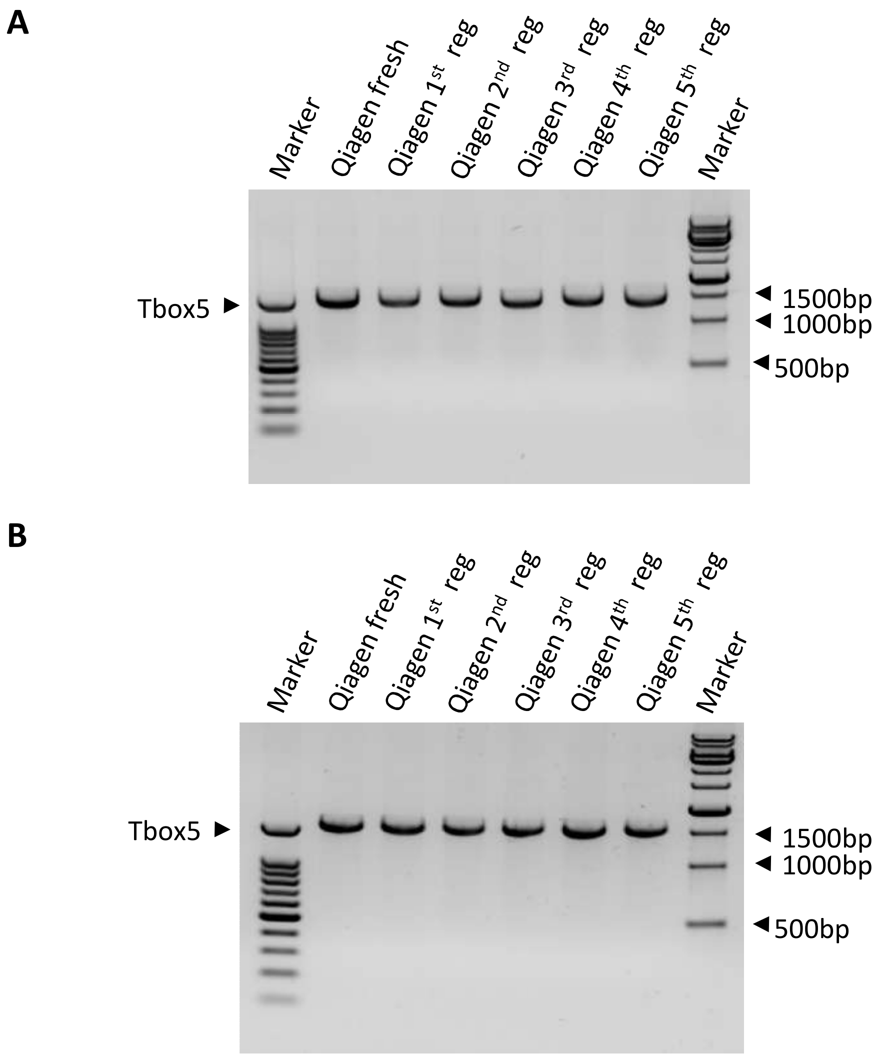


**Fig. S4. The used columns from Qiagen PCR purification and gel extraction kits can be repeatedly regenerated and reused a minimum of five times. A)** Analysis of DNA purification for the regenerated columns from Qiagen PCR purification kit. DNA-contaminated columns were cleaned using 1 M phosphoric acid and used for PCR product purification; the same columns were cleaned and reused four additional times. DNA concentration of the sample from each round was measured with Nanophotometer; the purified DNA was verified and imaged as shown in Fig. S2. **B)** Analysis of DNA extraction for the regenerated columns from Qiagen gel extraction kit. DNA-contaminated columns were repeatedly regenerated and reused as described in A, DNA extraction from agarose gel was performed according to gel extraction kit manual. The data were processed as shown in A.

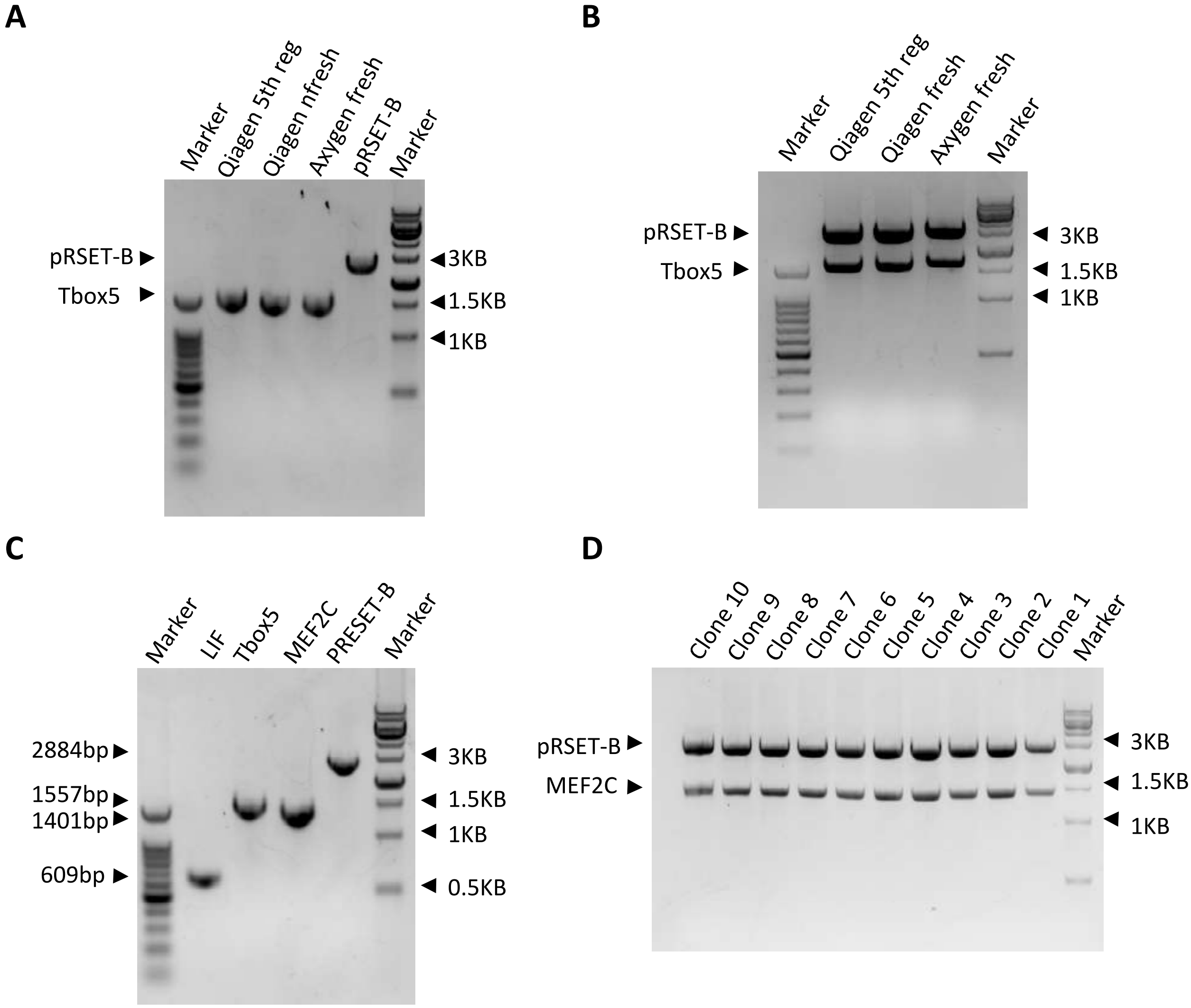


**Fig. S5. The DNA prepared with the regenerated columns dose not affect cloning efficiency.** A) A agarose gel electrophoresis image for the DNA samples purified with the regenerated or fresh columns. One microgram of DNA was digested with *Bam*H I and *Hind* III, followed by agarose gel electrophoresis and DNA extraction. B) A electrophoresis image for the positive clones digested with *Bam*H I and *Hind* III. The DNA purified with the fresh or regenerated columns was used for gene cloning. Fifty colonies were picked from LB plates for plasmid preparation. Positive clones were detected by digestion with *Bam*H I and *Hind* III, one positive clone for each sample was loaded into an agarose gel for electrophoresis. C) A electrophoresis image for the DNA samples purified with regenerated columns. Tbox5 DNA digested with *Bam*H I and *Hind* III was purified from gel with the regenerated columns. The Tbox5-contaminated columns were further regenerated using 1M phosphoric acid and used for purification of the MEF2C gene that were also digested with *Bam*H I and *Hind* III. The obtained DNA was detected by agarose gel electrophoresis. D) A electrophoresis image for MEF2C positive clones digested with *Bam*H I and *Hind* III. Positive clones were screened as described in B, 10 positive clone digested with restriction enzymes were loaded into an agarose gel for electrophoresis.
